# Narrative competence among hearing-impaired and normal-hearing children: analytical cross-sectional study

**DOI:** 10.1590/S1516-31802010000500008

**Published:** 2010-09-02

**Authors:** Alexandra Dezani Soares, Bárbara Niegia Garcia de Goulart, Brasilia Maria Chiari

**Affiliations:** I MSc. Speech-language pathologist, Hospital São Paulo, Universidade Federal de São Paulo (Unifesp), São Paulo, Brazil.; II PhD. Speech-language pathologist, Universidade Federal de São Paulo (Unifesp), São Paulo, and adjunct professor, Universidade Federal do Rio Grande do Sul (UFRGS), Porto Alegre, Rio Grande do Sul, Brazil.; III PhD. Full professor, Department of Speech-Hearing Sciences, Universidade Federal de São Paulo (Unifesp), São Paulo, Brazil.

**Keywords:** Hearing loss, Narration, Language disorders, Communication disorders, Speech-language pathology, Perda auditiva, Narração, Transtornos da linguagem, Transtornos da comunicação, Patologia da fala e linguagem

## Abstract

**CONTEXT AND OBJECTIVE:**

Oral narrative is a means of language development assessment. However, standardized data for deaf patients are scarce. The aim here was to compare the use of narrative competence between hearing-impaired and normal-hearing children.

**DESIGN AND SETTING:**

Analytical cross-sectional study at the Department of Speech-Language and Hearing Sciences, Universidade Federal de São Paulo.

**METHODS:**

Twenty-one moderately to profoundly bilaterally hearing-impaired children (cases) and 21 normal-hearing children without language abnormalities (controls), matched according to sex, age, schooling level and school type, were studied. A board showing pictures in a temporally logical sequence was presented to each child, to elicit a narrative, and the child’s performance relating to narrative structure and cohesion was measured. The frequencies of variables, their associations (Mann-Whitney test) and their 95% confidence intervals was analyzed.

**RESULTS:**

The deaf subjects showed poorer performance regarding narrative structure, use of connectives, cohesion measurements and general punctuation (P ≤ 0.05). There were no differences in the number of propositions elaborated or in referent specification between the two groups. The deaf children produced a higher proportion of orientation-related propositions (P = 0.001) and lower proportions of propositions relating to complicating actions (P = 0.015) and character reactions (P = 0.005).

**CONCLUSION:**

Hearing-impaired children have abnormalities in different aspects of language, involving form, content and use, in relation to their normal-hearing peers. Narrative competence was also associated with the children’s ages and the school type.

## RESUMO

**CONTEXTO E OBJETIVO:**

A narrativa oral constitui elemento de avaliação do desenvolvimento lingüístico, entretanto, são escassos os dados padronizados para pacientes surdos. O objetivo foi comparar o uso das competências narrativas entre crianças deficientes auditivas e ouvintes.

**TIPO DE ESTUDO E LOCAL:**

Estudo transversal analítico no Departamento de Fonoaudiologia, Universidade Federal de São Paulo.

**MÉTODO:**

Foram avaliadas 21 crianças com deficiência auditiva de grau moderado a profundo bilateral (casos) e 21 crianças ouvintes sem alterações de linguagem (controles) pareadas por sexo, idade, grau de escolaridade e tipo de escola frequentada. Foi apresentada uma prancha com figuras em sequência lógico-temporal para cada criança, para elicitar uma narrativa, e foi medido o desempenho na estrutura da narrativa e coesão. Foram analisadas as frequências das variáveis, suas associações (teste de Mann-Whitney) e intervalos de confiança de 95%.

**RESULTADOS:**

As crianças surdas apresentaram desempenho pior, na estrutura da narrativa, uso de conjunções, medidas de coesão e pontuação geral da narrativa (P ≤ 0,05). Não houve diferença no número de proposições elaboradas e especificação de referentes nos dois grupos. Os surdos produziram maior proporção de proposições do tipo orientação (P = 0,001) e menor proporção dos tipos ações complicadoras (P = 0,015) e reação do personagem (P = 0,005).

**CONCLUSÃO:**

Os deficientes auditivos possuem alterações nos diferentes aspectos da linguagem, envolvendo forma, conteúdo e uso em relação a seus pares ouvintes. A competência narrativa também está relacionada ao tipo de escola e idade das crianças estudadas.

## INTRODUCTION

The act of narration allows speech-language specialists to reach out to people and their stories, in order to meet their needs and concerns within the teaching-learning relationship. Narratives develop with early language acquisition during mother-child, social and family interactions, and at school as well. Narrative skills improve as children grow and develop and can be influenced by several cultural and linguistic factors.^1,2^ Narratives provide a rich linguistic context and have been used to evaluate the linguistic development of individuals with different health conditions that may be associated with language disorders.^3-14^

The interplay of hearing and language has a key role in ensuring quality of oral narrative as an expression of thought. Lack of access to appropriate and reliable instruments for evaluation of linguistic potential among hearing-impaired children may interfere with adequate speech and language therapy planning and thus compromise the effectiveness of speech-language interventions.^15^ Since few evaluation instruments that are properly validated for hearing-impaired patients are available in Brazil, their oral language is quite often subjectively evaluated, or it is evaluated using instruments that have not been properly validated for the Brazilian population. This may lead to unreliable evaluations of such patients’ communicative potential.^15^

## OBJECTIVES

To compare narrative competence between hearing-impaired speakers and normal-hearing children to provide input that will support this approach for speech and language evaluation and diagnosis of linguistic and pragmatic competence among hearing-impaired speakers; and to assess the association between narrative competence of hearing-impaired children and variables such as age, severity of hearing loss, age at diagnosis of hearing impairment, age at start of hearing aid use, length of speech and language therapy, and children’s schooling.

## METHODS

This analytical cross-sectional study was approved by the Research Ethics Committee of Hospital São Paulo (Protocol No. 1576/05). A total of 42 children (both males and females), aged 5 to 11 years old, were included in the study, of whom 21 had moderate to profound bilateral neurosensory hearing impairment (cases). These children had presented hearing loss onset prior to the age of three years, were users of individual hearing aids, and largely communicated using an oral linguistic code. The control group consisted of 21 children with normal hearing and no communication or learning disorders, who were matched with the cases according to age, gender, schooling level and school type (public or private).

Data on the children’s history were gathered through interviews with their mothers at the Laboratory of Communication Disorders Relating to Hearing Impairment, Department of Speech-Language and Hearing Sciences, Universidade Federal de São Paulo (Unifesp), Brazil.^16^ Narrative data were gathered in the same way in both groups studied. To elicit an oral narrative, a set of pictures from "The Dog’s Story," by Le Boeuf,^17^ was displayed on a single board with pictures properly arranged in sequence. The evaluator would tell the children under evaluation that these pictures made a story. They were asked to think how they would tell the story presented in the pictures and also to give a title to their story. All the children were told to take their time to familiarize themselves with the pictures before starting their narrative. While children were narrating their story they were allowed to make any visual contacts that they wished or to touch the board of pictures at any time. All the evaluations were recorded using a digital camera (Sony, Cybershot DSC-W30) and then saved in a computer for canonical speech transcription.

The analysis of narratives was performed by two independent speech-language pathologists with at least five years of clinical experience. It was based on narrative length and structure,^18^ and on cohesion measurements.^6^ The narrative length was measured by counting the total number of propositions, verbs and plots that were produced during each oral narrative.^18^ The narrative structure comprised the syntax and organization of its elements and was evaluated based on the following criteria.^18^

The propositions in the narratives were categorized, with regard to frequencies, as follows:

Orientation: propositions referring to setting and description of objects, characters and actions (a boy meets a lost dog on the street and takes it home and hides it in the closet);

Complicating actions: propositions referring to the sequence of events (a mother sees a dog in the closet and asks the boy what it is doing there);

Character’s reaction: propositions referring to the characters’ reactions to the events (the boy’s reaction when he found the dog or the mother’s reaction when she found the dog in the closet);

Resolution: sequence of propositions after the narrative reaches its culminating point (the boy’s mother allows him to keep the dog).

The classification of narrative structures was based on proposition scores.

A specific protocol was used to evaluate cohesion and reference elements.^18^ Narrative cohesion was measured by summing the proposition scores with the use of these elements. The overall narrative score for each group was obtained by summing the scores for narrative structure and cohesion measurement for each child studied.

The children’s ages, severity of hearing loss, age at diagnosis of hearing impairment, age at start of hearing aid use and length of speech and language therapy, and the children’s and mothers’ schooling levels were analyzed in association with narrative performance among the cases (hearing-impaired children) and the controls (normal-hearing children).

Statistical analysis was performed using the Mann-Whitney test to compare results between hearing-impaired and normal hearing children at a 5% level of significance. Frequencies, means, standard deviations and medians of the variables studied were also calculated, when applicable.

## RESULTS

A total of 42 children were evaluated, of whom 21 were hearing-impaired subjects (cases) and 21 were normal-hearing subjects (controls), as presented in Table 1. For the cases, hearing loss was diagnosed on average at the age of 38.8 months (standard deviation, SD = 26.9; median = 36 months). The mean age at which they started using hearing aids was 53.5 months (SD = 30.3; median = 60 months); and the mean length of their speech and language therapy was 41.9 months (SD = 34.4; median = 48 months). The average hearing loss in the better ear was 71.2 dB (SD = 18).

**Table 1.** Characteristics of the hearing-impaired children (cases) and normal-hearing children (controls)

Continuous variables	Hearing-impaired subjects (cases)		Normal-hearing subjects (controls)	
	Mean	Standard deviation	Mean	Standard deviation
Age (years)	8.2	1.6	8.2	1.6
Years of study	4.8	1.4	4.8	1.4
Mother’s schooling	10.9	4.3	10.9	4.3

Dichotomous variables	Hearing-impaired subjects (cases)		Normal-hearing subjects (controls)	
	n	%	n	%
Gender (male)	10	47.6	10	47.6
School type (private)	12	57.1	12	57.1

The type of school influenced the number of propositions used in the narrative (P = 0.04), especially regarding the use of propositions of resolution type (P = 0.00). Moreover, the age at evaluation also showed a significant relationship with oral narrative, for both groups (P = 0.03).

Table 2 presents the distribution of narrative propositions produced by the hearing-impaired and normal-hearing subjects in the sample studied.

**Table 2.** Comparison of narrative skills between hearing-impaired and normal-hearing children

Variables related to narrative skills	Hearing-impaired subjects (cases)		Normal-hearing subjects (controls)		P-value*
	Mean	Standard deviation	Mean	Standard deviation	
Number of propositions used	12.76	5.09	13.05	12.71	0.288
Score for narrative structure	3.14	1.77	4.43	0.93	0.004
Score for cohesion measurements	3.90	1.70	5.00	1.05	0.033
Overall narrative score	7.05	2.87	9.33	1.53	0.004

* Mann-Whitney test.

Tables 3 and 4 summarize the scores for the cohesion and reference elements among the hearing-impaired and normal-hearing subjects in the sample studied.

**Table 3.** Distribution of oral narrative propositions used by hearing-impaired and normal-hearing subjects

Types of proposition	Hearing-impaired subjects (cases)		Normal-hearing subjects (controls)		P-value*
	Mean %	Standard deviation	Mean %	Standard deviation	
Orientation	63.8	15.8	52.9	11.0	0.001
Complicating action	21.0	12.2	30.5	9.4	0.015
Character’s reaction	1.2	2.6	4.9	4.7	0.005
Resolution	10.0	7.5	11.8	5.7	0.614

* Mann-Whitney test.

**Table 4.** Scores for the cohesion elements and reference specifications among hearing-impaired and normal-hearing subjects

Variables	Hearing-impaired subjects (cases)			Normal-hearing subjects (controls)			P-value*
	Mean score	Median	Standard deviation	Mean score	Median	Standard deviation	
Cohesion elements	2.1	2.0	1.6	3.0	3.0	1.1	0.046
Reference scores	1.8	2.0	0.4	1.9	2.0	0.2	0.158

* Mann-Whitney test.

## DISCUSSION

The sample in this study presented sufficient statistical power (95%): 18 patients were needed for this study in each group, according to Compare 2 (WinPepi) with a sample size ratio of 1:1 and differences between means ranging from -0.54 to +0.54, and without any sample loss.

There are few studies in the literature analyzing narrative among hearing-impaired speakers.^13,14,18,19^ In the present study, the results from comparing narrative performance between hearing-impaired and normal-hearing children support the existence of an association between hearing and language for the development of narrative competence.

With regard to the different features used to analyze narrative competence, we found statistically significant differences in most of them, except for the number of propositions, reference score and use of resolution. These findings are consistent with data in other studies, which have reporting that children with language disorders produce fewer propositions than do children with normal language development.^6,7,12^

A longitudinal study investigating narrative development that included two children who were followed up from two to five years of age, without any history of language impairment,^20^ reported that at the age of two years, the children did not produce any narratives and only used temporal expressions in their vocabulary, along with the referent "now;"; at the age of three years, they started to use temporal relationships; at the age of 3-4 years, they started to develop stories in their discourse, with the introduction of markers such as "once upon a time" (to begin a narrative); "so," "then" and "after" (narrative operators); and "end of the story" and "happily ever after" (story closure).^20^ At the age of four years, the study children used "when" and direct speech. The author concluded that at that age, quality of discourse improves, but lack of cohesion may compromise the narrative quality. At the age of five years, the children became narrators and enunciators, and were able to communicate new information to adults.^20^

The hearing-impaired children in this study produced more orientation-related propositions while those with normal hearing produced more propositions of complicating actions and character reactions. In their oral narratives, hearing-impaired children more often used picture description and details, which would increase the number of orientation-related propositions and could interfere with the interlocutor’s understanding of the narrative.^18^

Regarding narrative structure, the hearing-impaired children had statistically significant lower performance scores, compared with normal-hearing children. Normal-hearing Brazilian Portuguese speakers with no impairments are able to elaborate full narratives from the age of five years.^20^ The lower scores for narrative structure among the hearing-impaired children in this study can be explained by delayed language development due to sensory deficiency, as reported in other studies,^8,16,21^ given that the subjects studied were five years of age and over.

Other authors^18^ have also described lower performance in relating to narrative structure among hearing-impaired children, compared with normal-hearing children. However, this difference was only statistically significant in relation to children with poor speech perception. It should be noted that, in the present study, oral communication was the main means of communication among the hearing-impaired children.

The hearing-impaired children in the present study had lower scores for the use of cohesion elements, but the difference was not statistically significant regarding reference use. Other authors have demonstrated lower use of cohesion elements in oral narratives among hearing-impaired individuals.^18^ Studies on other populations^3,4,9,22-28^ have also indicated that there is lower use of cohesion elements among individuals with language disorders.

In the present study, in comparison with the normal-hearing children, the hearing-impaired children had also lower scores for the cohesion measurements. In a study on written creation of a narrative based on pictures,^29^ lower rates of use of connectives, prepositions, pronouns, adverbs, verbs and determinants were found among hearing-impaired children than among normal-hearing children. These were also predictors of oral communication features.^29^

Narrative cohesion skills are well-developed around the age of seven years among children with normal development. Difficulties in using connectives may be associated with relational abnormalities and difficulties in integrating relationships with meanings, thereby resulting in separation of the story’s organization from the use of cohesion elements.^3,22^

Hearing-impaired children show delayed use of temporal/spatial notions in sentences, compared with normal-hearing children.^21^ In addition, semantic abnormalities seen in cases of hearing impairment^16^ may have contributed towards poor performance in the cohesion score.

The overall narrative score was expressed by the sum of narrative scores and cohesion measurement scores. It was seen that the hearing-impaired children had significantly lower narrative skills than those of the normal-hearing children. These differences in narrative performance corroborate previous findings among hearing-impaired individuals^18^ and other studies comparing individuals with language disorders and those without hearing impairment.^3,9,22^

The directly proportional association between age and the use of complicating actions in narratives is consistent with the findings of a previous study on children aged 7-8 years^24^ that found a strong association between age and cognitive and language measurements.

No previous studies investigating the association between narrative performance and school type (public or private) were found, thus suggesting that there is a need for further studies among hearing-impaired individuals, in order to provide more input on social and environmental factors that contribute towards the development of children and their communication skills, as reported in the literature.^1,19,24-26^

In contrast to other studies,^20,27,28^ we did not find any significant association between narrative performance and variables relating to the severity of hearing loss, such as age at diagnosis,^25,29^ age at start of hearing aid use,^30^ length of speech and language therapy, and children’s schooling. On the other hand, some other studies did not find an association between language measurements and severity of hearing loss,^25^ age at diagnosis and age at start of hearing aid use.^26^

It is noteworthy that narrative is a key instrument for evaluating linguistic skills among hearing-impaired individuals, since it enables identification of communication disorders that are not easily detectable in common tests and other instruments that are used for speech and language evaluation of oral communication, and it provides major qualitative input for speech and language planning.

## CONCLUSIONS

The present study showed that hearing-impaired children have abnormalities in all aspects of language: form, content and use. The abnormalities seem to be associated with these children’s inability to convert oral language-speech, as described in the literature. In addition, hearing-impaired speakers have inadequate narrative competence regarding the rate of proposition use, narrative scores, narrative cohesion, cohesion measurements and overall narrative scores. These are directly associated with the children’s ages and the type of school attended (public or private).
